# Flowering Buds of Globular Proteins: Transpiring Simplicity of Protein Organization

**DOI:** 10.1002/cfg.223

**Published:** 2002-12

**Authors:** Igor N. Berezovsky, Edward N. Trifonov

**Affiliations:** 1 Department of Structural Biology The Weizmann Institute of Science PO Box 26 Rehovot 76100 Israel; 2 Genome Diversity Centre Institute of Evolution University of Haifa Haifa 31905 Israel

## Abstract

Structural and functional complexity of proteins is dramatically reduced to a simple linear picture when the laws of polymer physics are considered. A basic unit of the protein structure is a nearly standard closed loop of 25–35 amino acid residues, and every globular protein is built of consecutively connected closed loops. The physical necessity of the closed loops had been apparently imposed on the early stages of protein evolution. Indeed, the most frequent prototype sequence motifs in prokaryotic proteins have the same sequence size, and their high match representatives are found as closed loops in crystallized proteins. Thus, the linear organization of the closed loop elements is a quintessence of protein evolution, structure and folding.


**Comparative and Functional Genomics**
*Comp Funct Genom* 2002; **3**: 525–534.
Published online in Wiley InterScience (www.interscience.wiley.com). **DOI:** 10.1002/cfg.223

**Conference Review**

### Flowering buds of globular proteins: transpiring simplicity of protein organization

Igor N. Berezovsky[1] and Edward N. Trifonov[2]*
[1] *Department of Structural Biology, The Weizmann Institute of Science, PO Box 26, Rehovot 76100, Israel*
[2] *Genome Diversity Centre, Institute of Evolution, University of Haifa, Haifa 31905, Israel*

**Correspondence to:
Edward N. Trifonov, Genome Diversity Centre, Institute of Evolution, University of Haifa, Haifa 31905, Israel.
E-mail:
trifonov@research.haifa.ac.il*

Received: 31 August 2002
Accepted: 14 October 2002

### Abstract

**Structural and functional complexity of proteins is dramatically reduced to a simple linear picture when the laws of polymer physics are considered. A basic unit of the protein structure is a nearly standard closed loop of 25–35 amino acid residues, and every globular protein is built of consecutively connected closed loops. The physical necessity of the closed loops had been apparently imposed on the early stages of protein evolution. Indeed, the most frequent prototype sequence motifs in prokaryotic proteins have the same sequence size, and their high match representatives are found as closed loops in crystallized proteins. Thus, the linear organization of the closed loop elements is a quintessence of protein evolution, structure and folding. Copyright © 2002 John Wiley & Sons, Ltd.**

**Keywords:** **loop closure; prototype elements; protein structure; protein folding; protein evolution; protein design; protein classification**

### Introduction

One fundamental property of the polypeptide chain is its ability to return to itself with the formation of closed loops. This phenomenon is enforced by the laws of polymer statistics, which also suggest a typical loop size, dependent only on chain flexibility [9,14]. For natural protein chains this closed loop size is about 25–35 amino acid residues [2]. Such loops are important elements of protein structure as is well documented in recent studies [2,3,4,10]. Every globular protein can be represented as a 1D to 3D assembly of closed loops of nearly standard size, as above. Until recently this basic feature of protein structure has never been explored. In the following review, this novel view on protein structure is outlined, and dramatic implications for protein folding and evolution are discussed.

### Chain returns — units of protein structure

The optimal size of the closed loops is a trade-off between the energy of deformation of the chain, and the probability of the loop ends occurring in the vicinity of one another. The loop closure fortified by the interactions between amino acid residues at the ends of the loops provides an important degree of stability, which makes the closed loop a distinct structural unit. Every loop may have its own unique conformation, with a specific arrangement of secondary structure elements along the contour of the loop and various intra-loop interactions.

### Disassembly of globular proteins into the closed-loop components

In a search for the closed loops as contours with the short C$\alpha$–C$\alpha$ contacts at the loop ends, one immediately discovers that practically every globular protein is built as a linear assembly of closed loops of nearly standard size [2,4], about 30 residues, compacted in the 3D globule. Several examples of such linear organization are illustrated in Figure 1. Here, the globules of the major fold types [12], placed in the centre of a respective Figure, are surrounded by the elementary closed loops following one after another clockwise according to their

Copyright © 2002 John Wiley & Sons, Ltd.

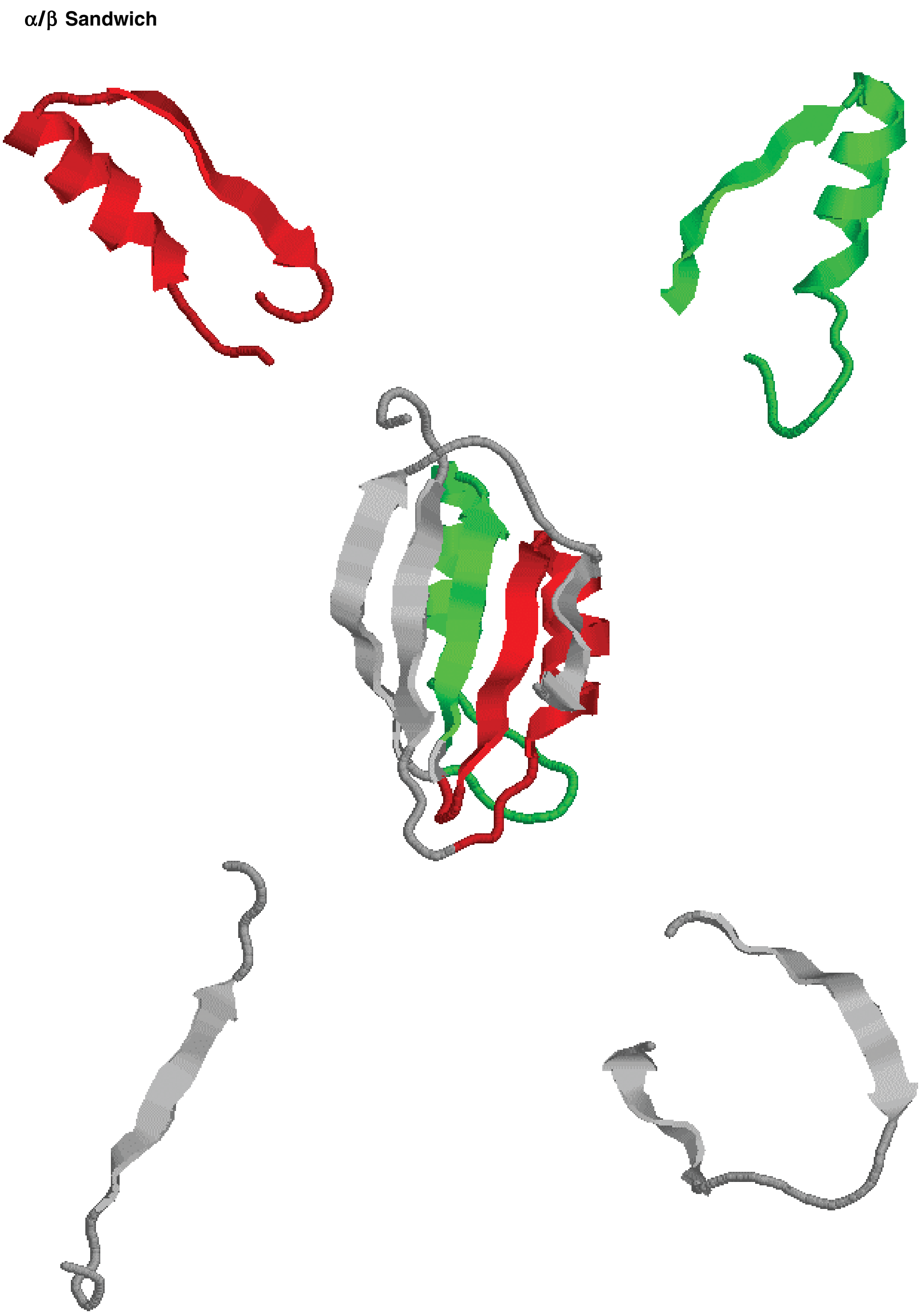

**Figure 1.** Representative examples of major folds [12] disassembled in closed loop elements. The coordinates of the loop ends are calculated from the shortest $C\alpha - C\alpha$ distances [2], accepting the contour length within the range 15–35 amino acid residues. ***αβ* Sandwich** (1aps): N-end segment (residues 1–17, grey); loop 1 (18–45, red); loop 2 (47–75, green); opened C-end loop (76–98, grey). **Trefoil fold** (1i1b): N-end segment (residues 1–13, grey); loop 1 (16–30, red); loop 2 (42–62, green); loop 3 (70–99, blue); loop 4 (101–115, orange); loop 5 (120–136, magenta); C-end segment (139–153, grey). **Doubly Wound** (2fox): loop 1 (1–30, red); loop 2 (35–66, green); loop 3 (78–107, orange); loop 4 (112–134, blue). **Jelly Roll** (2stv): N-end segment (residues 12–27, grey); loop 1 (29–58, red); loop 2 (72–89, green); loop 3 (93–124, blue); loop 4 (129–147, orange); loop 5 (154–170, magenta); loop 7 (171–190, cyan). **Immunoglobulin fold** (2rhe): loop 1 (3–24, red); loop 2 (29–52, green); loop 3 (53–74, blue); loop 4 (81–109, orange)

Copyright © 2002 John Wiley & Sons, Ltd. 

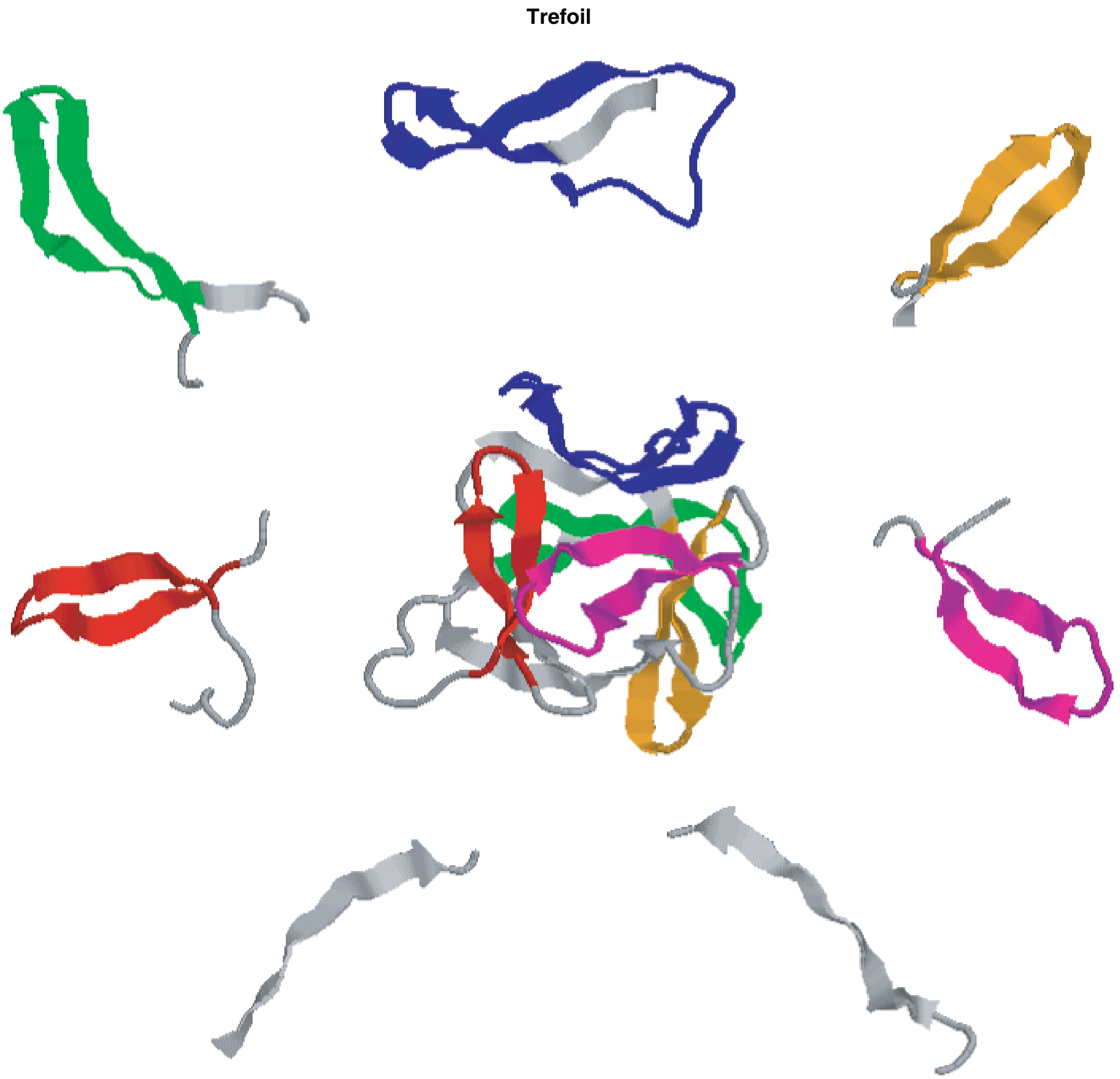

**Figure 1.** Continued consecutive engagement. Space limitations do not allow us to display all the diversity of existing fold types. However, these typical examples demonstrate the spectacular picture of the flowering buds (protein globules) with their petals (loops) exposed.

### From closed loops to folds

Inspection of the figures reveals that all different folds have the same organization. Naturally, specific interactions between the loops and details of the secondary structure of the closed loops would dictate their 3D arrangements in the variety of forms. Complicated as the forms appear, they are actually rather simple when looked at as an assembly of loops. One interesting example is the case of cytochromes *c* and 256*b*. These two functionally related proteins involved in electron transport have practically the same size (112 and 106 amino acid residues, respectively), and the same sequence positions of the loop ends. Their folds are, however, rather different (cytochrome *c* type, and four-helical up-and-down bundles, respectively, according to the SCOP classification [11]). As Figure 2 illustrates, the difference is essentially due to a

Copyright © 2002 John Wiley & Sons, Ltd. 

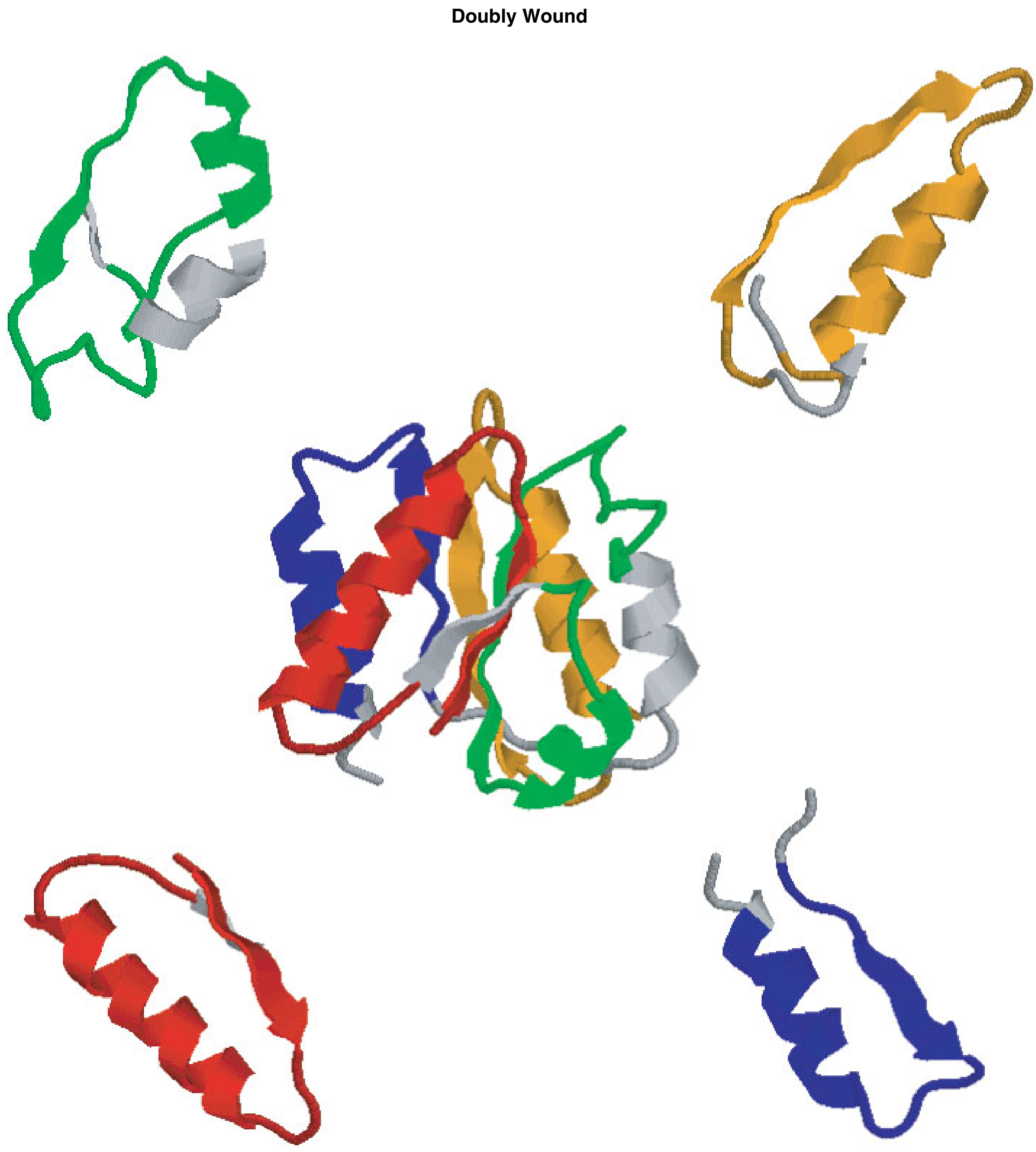

**Figure 1.** Continued change in orientation of the middle loop (green). In the cytochrome *c* structure this loop is 'head-up', while in the 256*b* structure it is 'head-down'. Several other changes are seen as well (see legend for the Figure). In the bottom of Figure 2 the two corresponding sets of the coloured protein sections are shown for comparison. Interestingly, after the apparent transformation the middle loop (green) almost did not change, whereas two other sections are rather disfigured. This example indicates that the closed loop organization is rather versatile, which opens an intriguing dimension in protein evolution and design.

A straightforward evolutionary scenario would be the formation of multiloop structures from a variety of different types of closed loops. Some

Copyright © 2002 John Wiley & Sons, Ltd. 

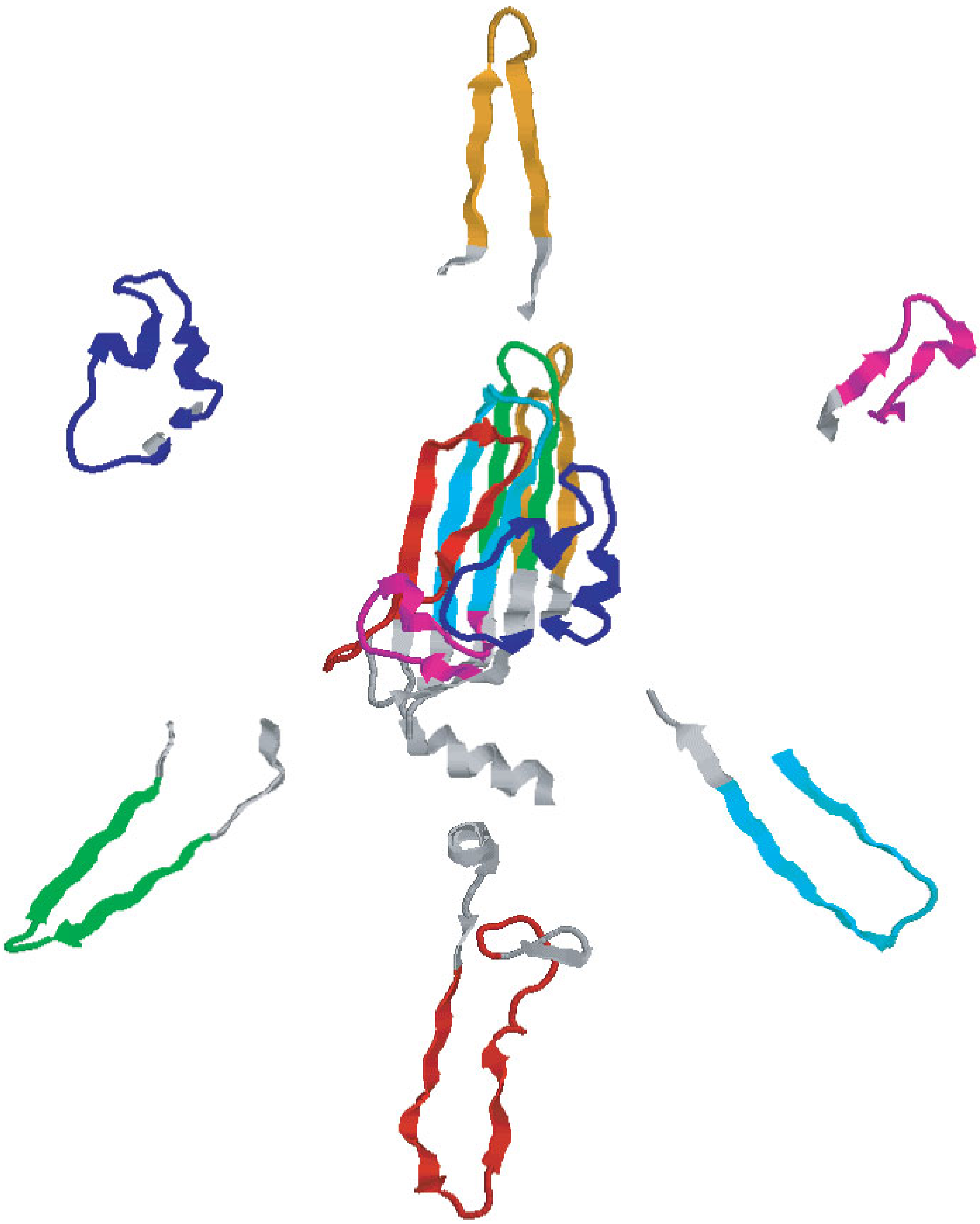

**Figure 1.** Continued

Copyright © 2002 John Wiley & Sons, Ltd.

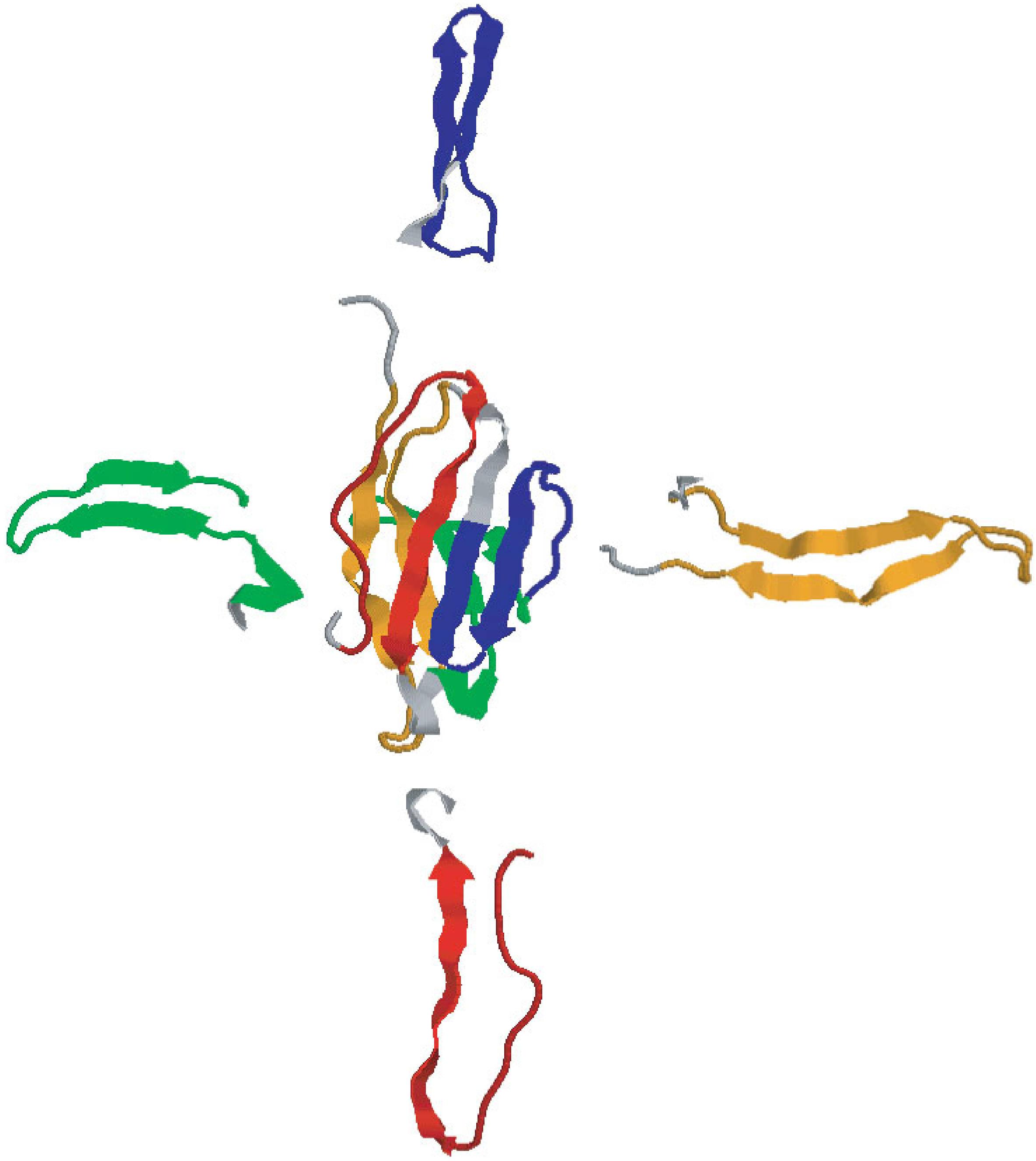

**Figure 1.** Continued of the types may well be present simultaneously in many related or only distantly related proteins. Probably the best illustration of such omnipresence is the so-called P-loop involved in ATP- and GTP-binding [13].

### Closed loops: evolutionary connection

When the protein chains evolved from short to long and reached a size adequate for loop closure, a new important evolutionary stage opened [15].

Copyright © 2002 John Wiley & Sons, Ltd. 

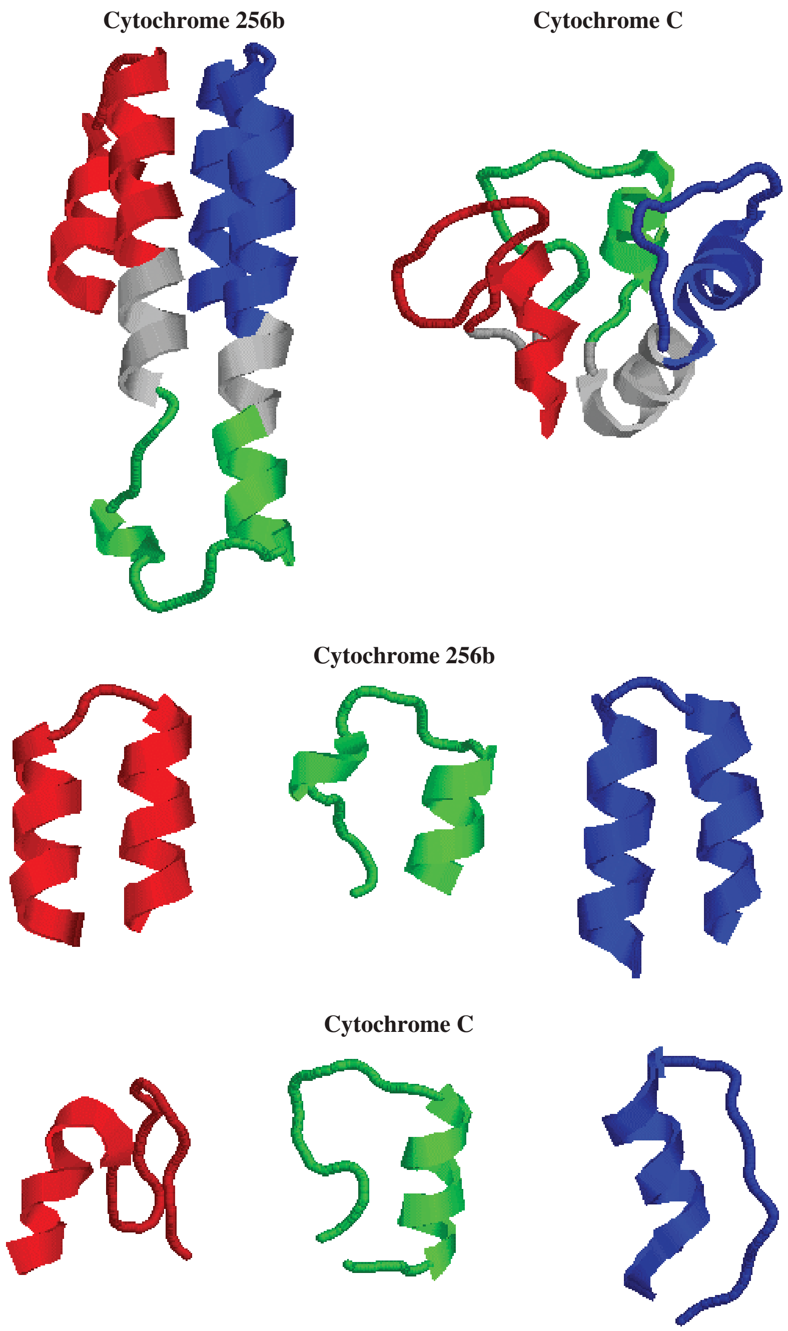

**Figure 2.** Comparison of two cytochrome structures. (Top) The cores of respective folds (in both cases residues 10–94). (Bottom) Two respective sets of the loops. Cytochrome 256*b*: loop 1 (10–33, red); loop 2 (41–62, green); loop 3 (68–94, blue). Cytochrome *c*: section 1 (10–33, red); loop 2 (39–61, green); loop 3 (72–94, blue)

The closure provided advantageous stability to the standard loop size molecules, and a whole population of different molecules of that size should have become dominant. During this evolutionary period, presumably, the functions associated with the individual closed loops were consolidated and optimized. Later, fusion of respective genes would have resulted in various combinations of the loops and their functions. Modern proteins may well keep this original organization, as it indeed appears

Copyright © 2002 John Wiley & Sons, Ltd. 

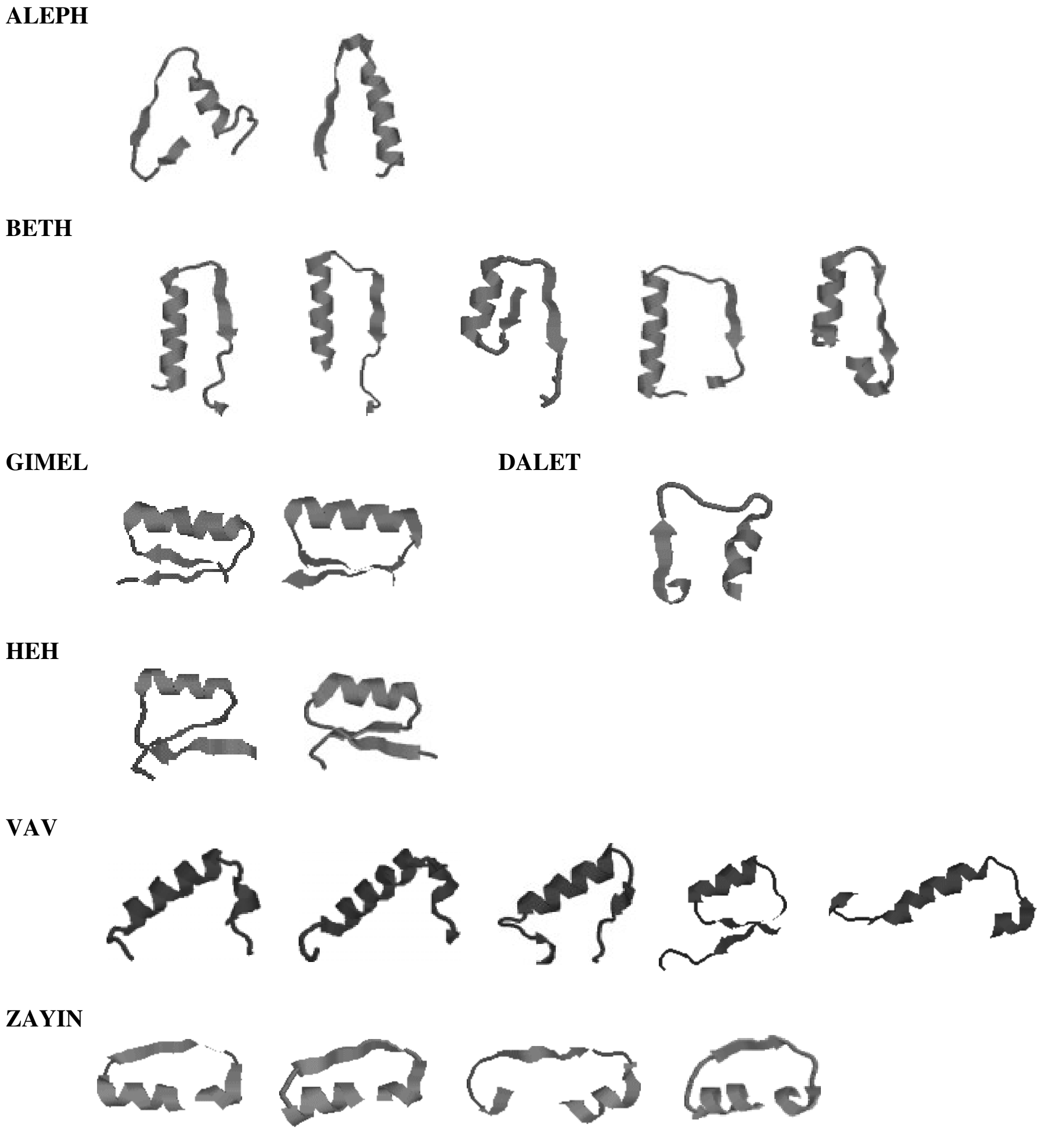

**Figure 3.** Gallery of highest match descendants of the seven sequence/structure prototypes (Aleph to Zayin) detected in the crystallized proteins

(see below). In that case the loop elements would be descendants of the original small molecules with their sequence/structure features, hopefully still conserved to a detectable degree. The original structural and functional identity of these elements may still play a role in later evolutionary recombination events. As suggested by the analysis of the positions of hot spots for recombinational swapping of protein sequence segments [18], the size of 20–30 amino acid residues corresponds to apparent protein building blocks.

A massive search for the most common sequence motifs of the size 25–35 amino acid residues in 23 prokaryotic proteomes yielded the above-mentioned P-loop motif as the most frequent. It is located in hundreds of proteins involved in a variety of energy-dependent processes. Several other frequent sequence motifs have also been detected [5,7,16,17]. Remarkably, their mapping on the sequences of crystallized protein structures revealed that the common motifs are located in the closed loops. In Figure 3 structural representatives

Copyright © 2002 John Wiley & Sons, Ltd.

(highest sequence matches) of seven families of the loops are shown. As they are presumably the most ancient forms, the respective sequence/structure prototypes are numbered according to the letters of the ancient Hebrew alphabet. The Aleph group corresponds to the P-loop located primarily in the ABC-transporter proteins [1]. The fact that at least some prototypes can be reconstructed demonstrates that billions of years of evolutionary changes have not completely destroyed the original patterns/structures. We expect that more prototypes will be found by further scrutiny of sequence and structure databases. Mapping of the prototypes onto protein sequences would give an important lead in analyses of functional and evolutionary relatedness of various proteins. One good example of such mapping [8] is the ATP-binding subunit of histidine permease from *S. typhimurium* (1b0u PDB code), where five of the seven above prototypes were located. Its structure could be presented as a 'word', X–Aleph–X–Dalet–Vav–Beth–Zayin–X, where the symbol X corresponds to the sections that cannot immediately be recognized as descendants of some other hypothetical prototypes. It may well be that the uncharacterized sections have rather recent *de novo* origin, so that no distinct prototype has yet developed. They are, however, under the same structural and physical pressures, requiring the same size scaling. The prototypes Vav (Figure 3, blue) and Zayin (Figure 3, magenta) were actually detected originally in this very protein as standard size loop elements, sandwiched between earlier-characterized prototype descendants.

### Concluding remarks

According to the view described above, the structurally and functionally complicated protein globules unravel into simple linear assemblies, just as flowering buds open, revealing their petals. The linearity of the organization of the closed loops is apparently rooted in early protein evolution. The linearity also has far-reaching implications for protein synthesis and folding [5,6]. An obvious scenario is sequential co-translational folding of the loops (their closure and formation of internal secondary structures), with subsequent arrangement of the loops in final 3D organization. The observed times required for protein folding are consistent with the existence of small (of the order of 20–50 amino acid residues), independently folding units [7]. The linearity rule makes the very basis of the evolutionary history of globular structures, of their organization as linear arrays of closed loops, and of their sequential (co-translational) folding during protein synthesis. The variety of 'petals' (closed loops) and their combination renders to protein structure all its natural beauty.

### References

1. Bairoch A, Bucher P, Hofmann K. 1997. The PROSITE database, its status in 1997. *Nucleic Acids Res* **25**: 217–221.
2. Berezovsky IN, Grosberg AY, Trifonov EN. 2000. Closed loops of nearly standard size: common basic element of protein structure. *FEBS Lett* **466**: 283–286.
3. Berezovsky IN, Trifonov EN. 2001a. Van der Waals locks: loop-n-lock structure of globular proteins. *J Mol Biol* **307**: 1419–1426.
4. Berezovsky IN, Trifonov EN. 2001b. Loop fold nature of globular proteins. *Protein Eng* **14**: 403–407.
5. Berezovsky IN, Trifonov EN. 2001c. Protein structure and folding: a new start. *J Biomol Struct Dyn* **19**: 397–403.
6. Berezovsky IN, Kirzhner A, Kirzhner VM, Trifonov EN. 2001. Protein folding: looping from the hydrophobic nuclei. *Proteins* **45**: 346–350.
7. Berezovsky IN, Trifonov EN. 2002. Loop fold structure of proteins: resolution of Levinthal's paradox. *J Biomol Struct Dyn* **20**: 5–6.
8. Berezovsky IN, Kirzhner A, Kirzhner VM, Trifonov EN. 2002. An eye-opener to protein structure. *ComPlexUs* (in press).
9. De Gennes PG. 1990. *Introduction to Polymer Dynamics*. University Press: Cambridge; 57.
10. Lamarine M, Mornon J-P, Berezovsky IN, Chomilier J. 2001. Distribution of tightened end fragments of globular proteins statistically match that of topohydrophobic positions: towards an efficient punctuation of protein folding? *Cell Mol Life Sci* **58**: 492–498.
11. Murzin AG, Brenner SE, Hubbard T, Chothia C. 1995. SCOP: a structural classification of proteins database for the investigation of sequences and structures. *J Mol Biol* **247**: 536–540.
12. Orengo CA, Jones DT, Thornton JM. 1994. Protein superfamilies and domain superfolds. *Nature* **372**: 631–634.
13. Saraste M, Sibbald PR, Wittinghofer A. 1990. The P-loop — a common motif in ATP- and GTP-binding proteins. *Trends Biochem Sci* **15**: 430–434.
14. Shimada J, Yamakawa H. 1984. Ring-closure probabilities for twisted wormlike chains. Application to DNA. *Macromolecules* **17**: 689–698.

Copyright © 2002 John Wiley & Sons, Ltd. 

15. Trifonov EN, Kirzhner A, Kirzhner VM, Berezovsky IN. 2001. Distinct stages of protein evolution as suggested by protein sequence analysis. *J Mol Evol* **53**: 394–401.
16. Trifonov EN, Berezovsky IN. 2002a. Proteomic code. *Mol Biol* **36**: 239–243.
17. Trifonov EN, Berezovsky IN. 2002b. Molecular evolution from abiotic scratch. *FEBS Lett* **527**: 1–4.
18. Voigt CA, Martinez C, Wang ZG, Mayo SL, Arnold FH. 2002. Protein building blocks preserved by recombination. *Nature Struct Biol* **9**: 553–558.

Copyright © 2002 John Wiley & Sons, Ltd. 
